# Risk factors of pneumothorax in computed tomography guided lung nodule marking using autologous blood: a retrospective study

**DOI:** 10.1186/s13019-024-02810-y

**Published:** 2024-06-01

**Authors:** Shaohang Wu, Jianyang Wu, Junkai Xiong, Chengbin Huang, Yiwei Lin, Jun Guan, Jianxin Xu

**Affiliations:** Department of Thoracic Surgery, The First Hospital of Putian, The School of Clinical Medicine, Fujian Medical University, No. 449 Nanmenxi Road, Putian, Fujian 351100 China

**Keywords:** Pulmonary nodules, Autologous blood localization, Complications

## Abstract

**Background:**

To investigate the risk factors of pneumothorax of using computed tomography (CT) guidance to inject autologous blood to locate isolated lung nodules.

**Methods:**

In the First Hospital of Putian City, 92 cases of single small pulmonary nodules were retrospectively analyzed between November 2019 and March 2023. Before each surgery, autologous blood was injected, and the complications of each case, such as pneumothorax and pulmonary hemorrhage, were recorded. Patient sex, age, position at positioning, and nodule type, size, location, and distance from the visceral pleura were considered. Similarly, the thickness of the chest wall, the depth and duration of the needle-lung contact, the length of the positioning procedure, and complications connected to the patient’s positioning were noted. Logistics single-factor and multi-factor variable analyses were used to identify the risk factors for pneumothorax. The multi-factor logistics analysis was incorporated into the final nomogram prediction model for modeling, and a nomogram was established.

**Results:**

Logistics analysis suggested that the nodule size and the contact depth between the needle and lung tissue were independent risk factors for pneumothorax.

**Conclusion:**

The factors associated with pneumothorax after localization are smaller nodules and deeper contact between the needle and lung tissue.

## Background

Lung cancer is the most common cause of cancer death in men and women. Owing to the widespread use of lung cancer screening programs, thoracic surgeons currently encounter an increasing number of patients with isolated nodules in the lungs. The widespread use of high-resolution and thin-layer computed tomography (CT) has enabled the detection of significantly small lung nodules [1]. CT reveals early lung tumors usually as pulmonary nodules (PNs) [2–4]. Early diagnosis and treatment of this PN type are usually performed through video-assisted thoracoscopic surgery (VATS) [5–7]. Many small nodules are pathologically confirmed to be cancerous during removal [8]. The accurate localization of small PNs and ground glass nodules remains challenging in thoracoscopic surgery, and effective preoperative positioning can help thoracic surgeons quickly locate tumor nodules during surgery [9]. In addition, liquid material (methylene blue, iodide oil, medical glue, barium sulfate, radionuclide, and autologous blood), solid material, and other (ultrasonic positioning and near-infrared scanning) positioning technologies can be effective [10, 11]. Regarding the preoperative positioning technology for PN, the most reported invasive preoperative positioning methods are CT-guided Hook-wire and micro-spring ring positioning [12]. Menginghua et al. [13] reported that the total incidence of complications was 48.04% in the Hook-wire group and 33.85% in the micro-coil group, including pneumothorax, pulmonary hemorrhage, and chest pain. Previous studies [14] and Wu Gao’s report [15] demonstrated CT-guided injection of autologous blood to locate small PNs as a simple, intuitive, effective, and economical method. In this study, autologous blood was used for preoperative PN localization, and the complications and risk factors associated with CT-guided autoblood localization of small PNs were analyzed. The complications usually included pneumothorax, pulmonary hemorrhage, hemoptysis, and pain.

## Methods

### General information

This is a retrospective cohort study. Patients with isolated PNs located through the injection of autologous blood before VAST between November 2019 and March 2023 in the First Hospital of Putian City were included. There is no current unified standard for indicating PN puncture location; the process is primarily based on the comprehensive deduction of the nodule type and the distance from the pleura. Participants met the following criteria: ① solid nodule with a major axis diameter ≤ 1.5 cm and distance from visceral pleura ≥ 0.5 cm; ② pure ground glass nodules; and ③ the diameter of the main axis of the mixed grinding glass nodule was ≤ 2 cm and the distance from the pleura was ≥ 0.5 cm. The exclusion criteria were as follows: presence of ① pulmonary vascular lesions or lesions close to pulmonary large vessels; and ② severe cardiopulmonary insufficiency or bleeding tendency. Finally, 92 patients (44 males and 48 females) were enrolled in this study and were aged 54 ± 12 (range: 23–80) years. This study was approved by the Institutional Ethics Committee. All patients signed informed consent before positioning and VATS.

The data collected included demographic information, imaging features of the nodules (nodule nature, nodule size, nodule location, shortest distance from visceral pleura, and pleural depression sign), laboratory indicators before localization (forced vital capacity[FVC], forced expiratory volume in the first second/forced vital capacity[FEV1/FVC], maximal voluntary ventilation[MVV], white blood cell[WBC], and emphysema), position at localization, depth of needle insertion, depth of needle to lung tissue contact, contact time, the duration of localization surgery, the complications of localization surgery, operation mode, operation time, and pathological diagnosis.

Successful definition of the marker is the presence of a visible autoblood stain on the visceral pleura at the time of thoracoscopy. Successful localization was defined as the localization of nodules using staining marks with/without autoblood stains or the guidance of pinholes on the visceral pleura, dissection, and radiographic excision in excised specimens.

### CT-guided autologous blood localization

**Fig. 1:**
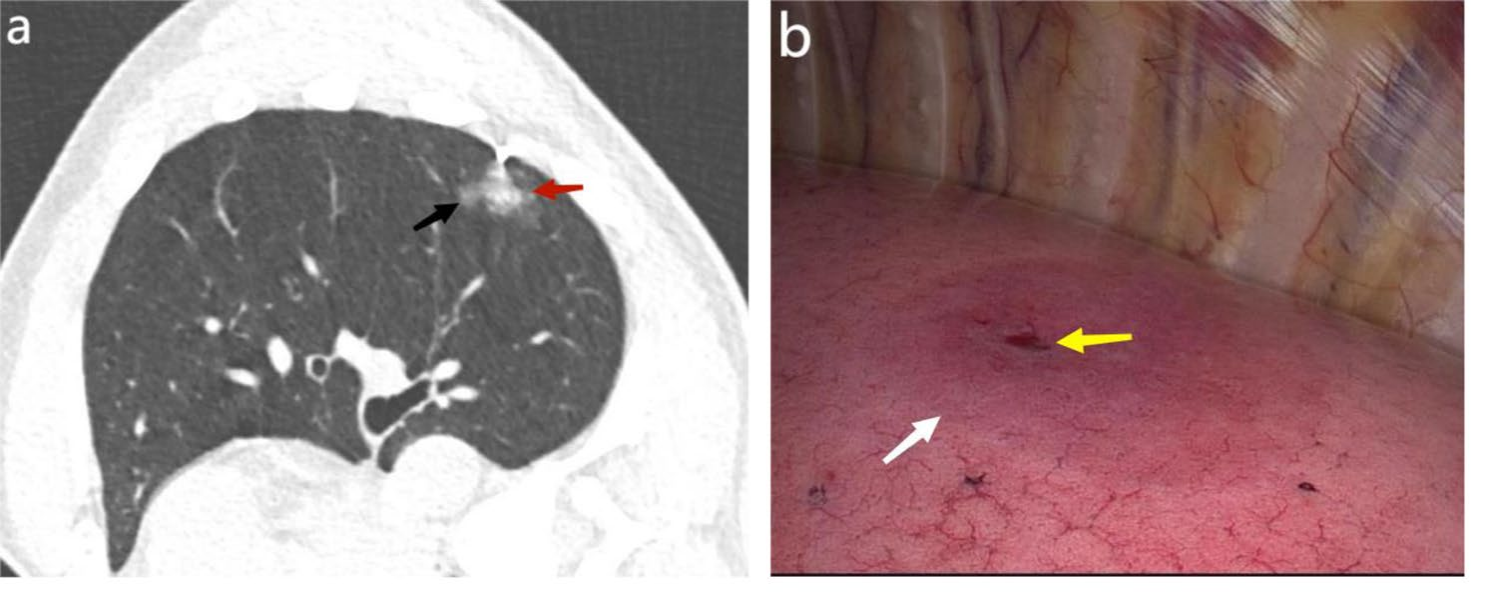
Computed tomography (CT)-guided autologous blood injection to locate isolated pulmonary nodules and intraoperative images. **A**. Chest CT shows a pure ground-glass nodule with a diameter of 0.7 cm in the left inferior lobe of the lungs (black arrow). In the patient’s lateral position, a CT-guided lung puncture was performed to inject autologous blood within 1 cm of the lesion (red arrow). **B**. Intraoperative pinholes (yellow arrows) and focal blood stains (white arrows) are seen under thoracoscopy

According to the preoperative imaging examination, the appropriate position was selected, and the distance between the nodules and the chest wall and parietal pleura was measured using chest CT thin-slice scanning(GE Healthcare, USA). Subsequently, the puncture site, needle insertion level, and needle insertion path were determined. Next, 5 mL of the patient’s autologous blood was extracted before using a puncture. After local lidocaine anesthesia, A percutaneous lung puncture was performed with a 20G puncture needle produced by CareFusion(address: Zona France las Americas, km,22-E-1. Santo Domingo. Dominican Republic) under the cooperation and guidance of radiology technicians. The shape path of the needle tip was adjusted according to timely CT 3D guidance, and it was finally located in the normal lung parenchyma approximately 1 cm around the small nodule. Autologous blood was injected next to the lesion, and the needle was gradually withdrawn. Furthermore, autologous blood was injected into the lung parenchyma through the path to the subpleural area (distance from pleura < 1 cm). After completion, CT was repeated to confirm the positioning effect and rule out complications such as pneumothorax and hemothorax (Fig. 1a). After localization, the patient returned to the ward and underwent thoracoscopic surgery the same day or the next day.

### Statistical analysis

Demographic information, imaging features of nodules, and data related to localization surgery of small PNs in thoracic surgery before autologous blood localization were retrospectively analyzed, and the complications of localization surgery in each group were recorded, including pneumothorax, pulmonary hemorrhage, hemoptysis, and pain. SPSS25.0 software was used for the statistical analysis. The Shapiro–Wilk test was used to evaluate normality. Continuous variables are expressed as mean and standard deviation (SD) or median and interquartile range(IQR), and counting data are expressed as frequency (n) and percentage (%). Logistics regression was used for single-factor analysis, and potential risk factors with *P* < 0.2 were obtained. Multivariate analysis was conducted using logistics regression to determine independent risk factors for pneumothorax. *P* < 0.05 was considered to be a statistically significant difference. The multi-factor logistics analysis was incorporated into the final nomogram prediction model for modeling. The identification and calibration of the model were evaluated. The bootstrap method was applied for further internal verification, and a nomogram was established.

### Ethical review

This study was reviewed by the Ethics Committee of Putian First Hospital. All patients signed informed consent before the surgery.

## Results

PN localization was successful in 89 cases (96.7%), Such as Fig. 1b, and failed in 3. All PNs were successfully removed by VATS without any conversion to thoracotomy. The general clinical data of patients are shown in Table 1. There were 92 nodules (44 males and 48 females; age 54 ± 12 [range: 23–80] years). There were 62 and 30 nodules in the non-inferior and inferior lobes, respectively. There were 12 solid, 77 ground glass, and 3 cystic nodules. The median nodule diameter was 0.9 cm (range: 0.5–2.0 cm). The median vertical distance between the nodules and the nearest pleura was 1.2 (range: 0–4.0) cm. FVC was 3.042 ± 0.772 L, FEV1/FVC was 85.755 ± 10.265%, MVV was 92.847 ± 26.403 L/min, and the median WBC was 6.01 (range: 3.15–10.74) E9/L. The median needle tip distances from the skin and the pleura were 6.3 (range: 3.6–11.2) and 2.2 (range: 0.9–4.6) cm, respectively. The median contact time between the puncture needle and lung tissue was 5 (range: 2–26) min, and the median total positioning time was 18 (range: 7–51) min. Sixteen cases (17.3%) had mild pneumothorax (the lung collapse rate in patients with pneumothorax was less than 20%) and no dyspnea; therefore, no indignant chest tube was placed. Two cases (2.0%) had mild pulmonary hemorrhage (found under thoracoscopic vision). However, there were no evident manifestations of hemoptysis; therefore, it was not identified before VAST, and there was no special treatment. All patients walked back to the ward without significant pain. The overall complication rate was 17.3% (16 of 92) (two patients had concurrent mild pneumothorax and pulmonary hemorrhage), and there were no serious complications.

**Table 1:** Patient and nodule characteristics

Clinical characteristic(*n* = 92)	Value *n*(%)
Age, y	
Mean ± SD	54 ± 12
Sex, n	
Male	44(48%)
Female	48(52%)
Solidity	
GGN	77(84%)
Solid	12(13%)
Cystic	3(3%)
Nodule size, cm	
Median (IQR)	0.9 (0.8, 1.2)
Distance, cm	
Median (IQR)	1.2 (0.4, 2)
FVC	
Mean ± SD	3.0 ± 0.8
FEV1/FVC	
Mean ± SD	85.8 ± 10.3
MVV	
Mean ± SD	92.8 ± 26.4
WBC	
Median (IQR)	6.0(5.3, 7)
Lung depth^a^, cm	
Median (IQR)	2.2(2, 2.6)
Insertion depth^b^, cm	
Median (IQR)	6.3(5.6, 7.2)
Puncture time, min	
Median (IQR)	5(3, 7.3)
Positioning time, min	
Median (IQR)	18(13, 25)
Emphysema	
Yes	22(24%)
No	70(76%)
Pleural stretch sign	
Yes	17(18%)
No	75(82%)
Location	
No-lower lobe	62(67%)
Lower lobe	30(33%)
Position	
Supine position	10(11%)
Prone position	20(22%)
Lateral position	62(67%)
Pneumothorax	
Yes	16(17%)
No	76(83%)
Surgery duration	
Mean ± SD	190.6 ± 78.2

^a^depth of the needle to lung tissue contact; ^b^depth of needle insertion.GGN, Ground-glass nodule; FVC, forced vital capacity; FEV1/FVC, forced expiratory volume in the first second/forced vital capacity; MVV, maximal voluntary ventilation; WBC, white blood cell; SD, standard deviation; IQR, interquartile range

**Fig. 2:**
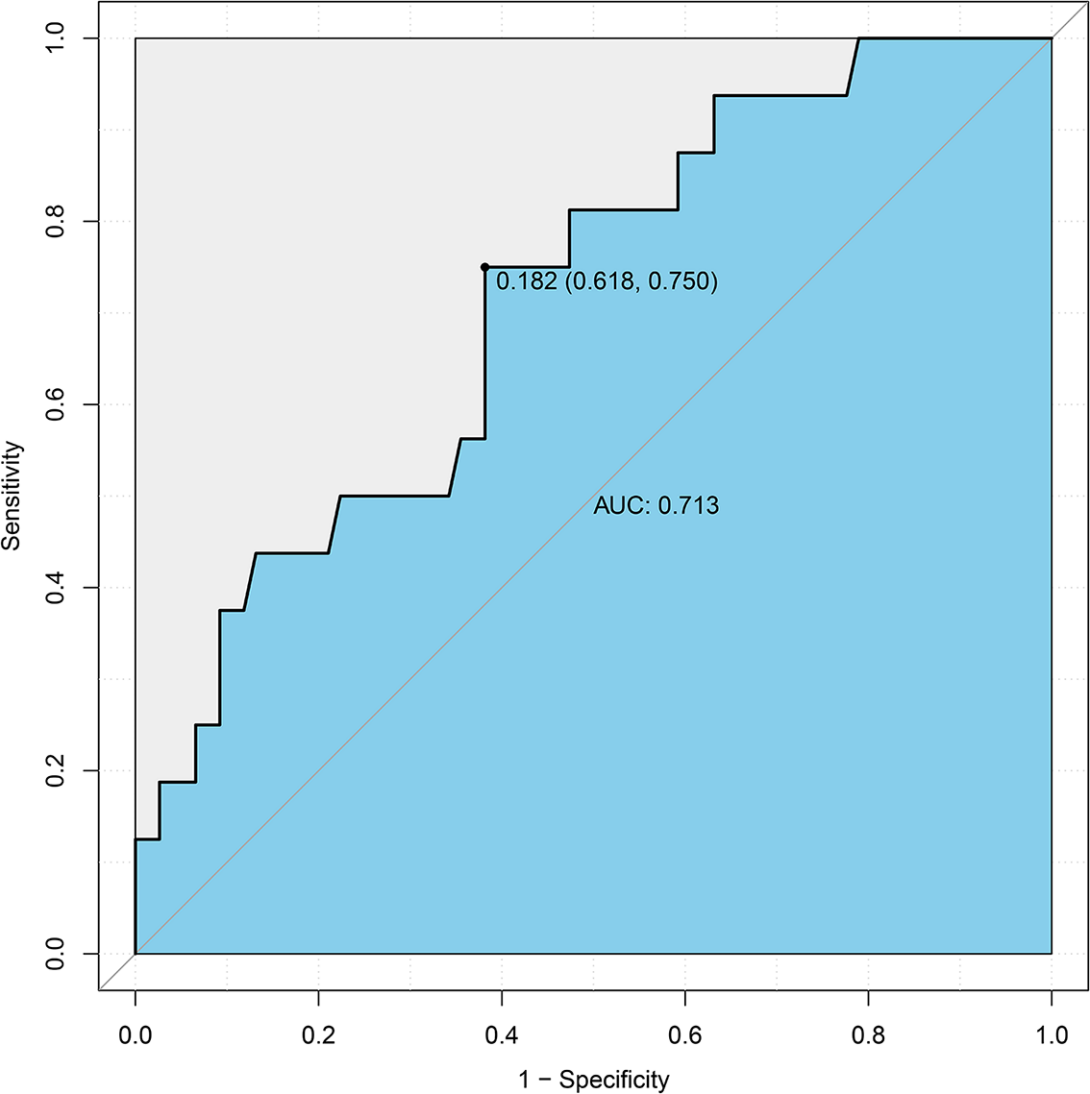
Receiver operating characteristic curve analysis was performed using puncture needle penetration depth and nodule size as independent variables and pneumothorax as dependent variables. The area under the curve was 0.713 (95% confidence interval = 0.618–0.750)

**Fig. 3:**
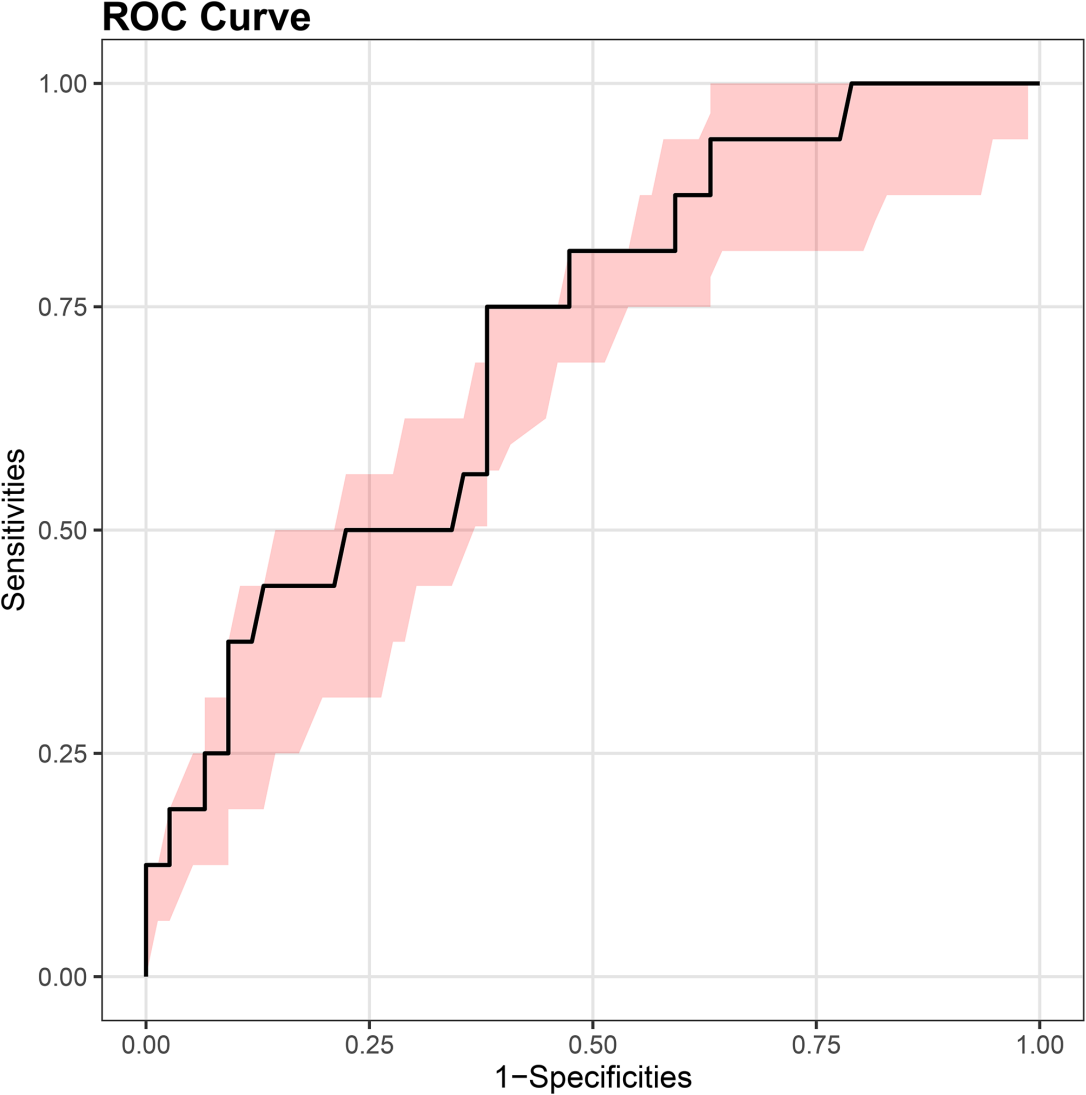
Receiver operating characteristic curve analysis was performed with internal validation (bootstrap method), and the area under the curve was 0.713 (95% CI = 0.603–0.720)

**Fig. 4:**
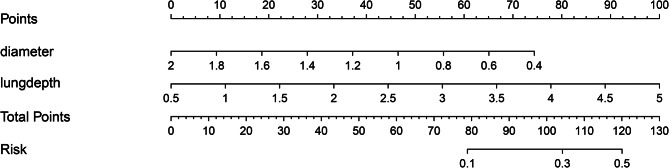
Nomogram for predicting the probability of autologous blood localization of isolated pulmonary nodules with pneumothorax. A score is assigned to the depth of needle contact with lung tissue and the diameter of the nodule by drawing a line upward from the corresponding value to the “dot line.” The “total score” is calculated as the sum of the individual scores for each of the two variables included in the column chart

**Fig. 5:**
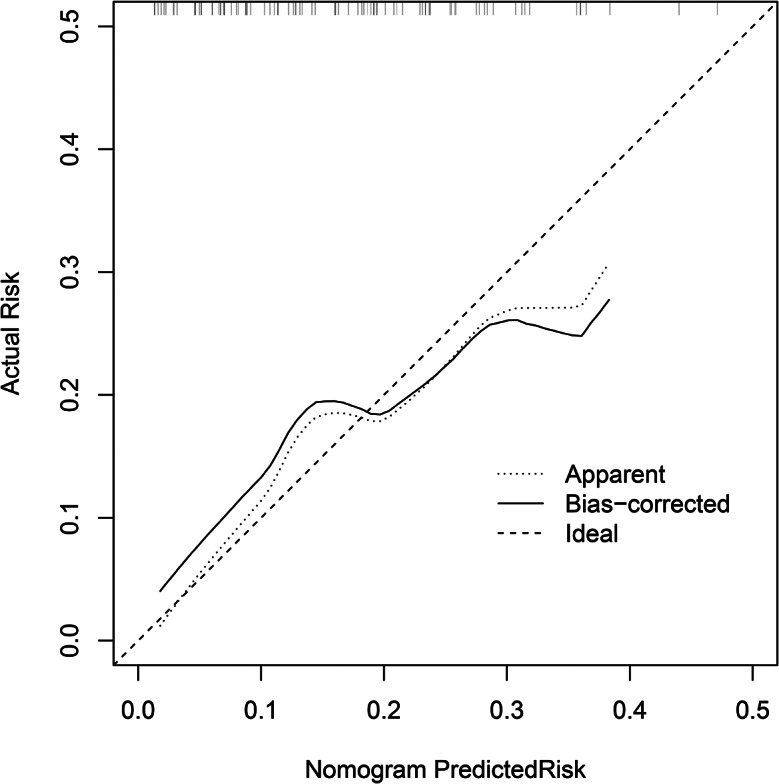
Calibration diagram of nomogram prediction model. The first dotted line represents the curve of the model, the second solid line is the corrected curve of the model, and the third dotted line represents the reference line where the ideal model is located

Single-factor analysis showed that FVC, MVV, WBC, depth of needle-to-lung tissue contact, tumor size, and nodule nature were the risk factors of pneumothorax in PNs located using autologous blood. Multivariate logistic regression analysis was used to study the risk factors for pneumothorax. The results showed that the depth of needle-to-lung tissue contact (odds ratio (OR) = 2.80; 95% confidence interval (Cl): 1.04–7.56; *P* = 0.042) and nodule size (OR = 0.04; 95%Cl: 0.00–0.51; *P* = 0.014) are the independent risk factors for locating isolated PNs complicated by pneumothorax induced by injecting autologous blood under CT guidance (Table 2). Receiver operating characteristic (ROC) curve analysis was performed using puncture needle penetration depth and nodule size as independent variables and pneumothorax as the dependent variable. Subsequently, a nomogram prediction model was established, and the area under the curve (AUC) was 0.713 (95% confidence interval = 0.618–0.750) (Fig. 2), indicating that the prediction ability of the model was acceptable. The bootstrap method was applied for internal verification. The AUC was 0.713 (95% confidence interval = 0.603–0.720) (Fig. 3), and a column graph was drawn (Fig. 4). The calibration diagram shows that the model has good prediction accuracy (Fig. 5).

**Table 2:** Univariate and multivariate analyses

Variables	Univariable		Multivariable	
	OR (95%CI)	*P* value	OR (95%CI)	*P* value
Year	1.00 (0.96–1.05)	0.907		
Puncture time	1.05 (0.91–1.20)	0.518		
FVC	0.61 (0.29–1.28)	0.190	0.81 (0.18–3.58)	0.780
FEV1/FVC	0.99 (0.94–1.04)	0.688		
MVV	0.98 (0.96–1.01)	0.134	0.98 (0.94–1.03)	0.418
WBC	1.33 (0.96–1.84)	0.082	1.42 (0.97–2.07)	0.068
Distance	1.25 (0.77–2.03)	0.372		
Insertion depth^b^	1.21 (0.90–1.64)	0.203		
Lung depth^a^	2.33 (1.04–5.20)	0.039	2.80 (1.04–7.56)	0.042
Tumor size	0.23 (0.03–1.56)	0.133	0.04 (0.00–0.51)	0.014
Positioning time	1.01 (0.95–1.07)	0.774		
Sex				
Male	Ref			
Female	0.66 (0.22–1.97)	0.460		
Emphysema				
No	Ref			
Yes	1.07 (0.31–3.75)	0.911		
Pleural stretch sign				
No	Ref			
Yes	1.02 (0.26–4.07)	0.975		
Nodule				
GGN	Ref		Ref	
Solid	0.45 (0.05–3.77)	0.460	0.45 (0.05–4.46)	0.494
Cystic	9.85 (0.83–116.80)	0.070	1.61 (0.10–26.92)	0.742
Location				
No-lower lobe	Ref			
Lower lobe	1.30 (0.42–3.99)	0.647		
Position				
Supine position	Ref			
Prone position	1.06 × 10^4^ (0.00–Inf)	0.990		
Lateral position	1.02 × 10^4^ (0.00–Inf)	0.990		

^a^depth of needle to lung tissue contact; ^b^depth of needle insertion; OR, odds ratio.GGN, Ground-glass nodule; FVC, forced vital capacity; FEV1/FVC, forced expiratory volume in the first second/forced vital capacity; MVV, maximal voluntary ventilation; WBC, white blood cell

PN localization was successful in 89 cases (96.7%) and failed in 3. Owing to extensive pulmonary adhesion, there were no evident blood stains on the surface of the visceral pleura after autologous blood injection, and no evident pinholes on the parietal and visceral pleura were observed in these three patients. In the other four cases, owing to extensive anthrax pigmentation, no evident blood stain was observed; however, the nodules could be resected successfully by guiding the pinholes in the parietal and visceral pleura. The operation time was 190.6 ± 78.2 min. Postoperative pathology showed that among the 92 nodules, six cases were inflammatory lesions, 1 was tuberculosis, 1 was alveolar epithelial hyperplasia, 1 was sclerosing pulmonary cell tumor, 1 was leiomyoma, 1 was a hamartoma, 4 were atypical adenomatoid hyperplasia, 3 were in situ adenocarcinoma, 31 were micro-invasive adenocarcinoma, 42 were invasive adenocarcinoma, and one was poorly differentiated.

## Discussion

Herein, we investigated the risks and side effects of using CT guidance to inject autologous blood to locate isolated lung nodules. Smaller nodules and deeper contact between the needle and lung tissue were found to be the factors associated with pneumothorax after localization.

In thoracoscopic surgery, the absence of preoperative localization increases the possibility of failed direct palpation of small PN. Therefore, locating such lesions is essential. Common localization methods include CT-guided indentation of locators in the lungs, such as wire with hooks, micro coils, methylene blue, and glue. Each method has its advantages and disadvantages. The hook wire is the most used and easy to operate. However, patients experience pain, a high incidence of complications, such as pneumothorax and intrapulmonary hemorrhage, and superficial lesions are prone to dislocation [16]. Microcoil has fewer complications and a good positioning effect [17, 18]; however, its operation is slightly complicated, and its consumables are more expensive. Biogel injections can cause coughing. If the injection site is too close to or within the lesion, Biogel may overwhelm the lesion and affect the pathological Sect. [19]. Injection of methylene blue and other liquid dyes is simple and economical. Its limitation is that the dye diffuses easily, resulting in poor positioning accuracy [20]. Prompt surgery should be performed after dye localization to avoid expanding the excision scope. The injection of iodide and barium provides a cheap and safe labeling method; however, fluoroscopic guidance is required during VATS, during which special equipment and radiation protection are required [21], increasing the radiation dose to patients and medical staff [22, 23]. Furthermore, barium may cause inflammation of surrounding lung tissue and affect the pathological diagnosis of nodules. Therefore, the above positioning methods have certain limitations.

Pneumothorax and pulmonary hemorrhage are common complications of CT-guided lung puncture [24, 25]. The overall complication rate of CT-guided lung puncture is reportedly 9–54% (average, 20%). Pneumothorax and intrapulmonary hemorrhage are common, and the incidence of critical complications requiring surgical treatment is less than 5% [26, 27]. Ye et al. reported that the incidence of pneumothorax was 31.9% while using medical glue to locate PN, compared with 51.4% during hook wire positioning. In addition, pulmonary parenchymal hemorrhage occurred in 13.9% of PNs treated with medical glue, compared with 13.5% of those treated with hook wire placement [28]. In this study, the incidence of pneumothorax was 17.3% and that of pulmonary hemorrhage was 2.2%. Among them, the degree of pulmonary collapse in 10 cases was 5–20% (two were found in VATS combined with a small amount of thoracic hemorrhagic fluid, two were owing to needle compression, and four were less than 5%), all of which were minor, required no special treatment, and exhibited no critical complications. Considering that the autologous blood coagulation rate is fast, it can effectively block the puncture site and reduce complications such as lung leakage and bleeding. According to Filippo’s study, an autologous blood clot is used as a blood patch, in which fine needle aspiration of PNs has a lower incidence of pneumothorax when there is bleeding around the needle path or high local bleeding (> 6 mm) [29]. In this study, the maximum diameter of blood distribution on CT images after autologous blood injection was 15.5 ± 5.7 mm, which may reduce the possibility of pneumothorax occurrence. Notably, because autologous blood is not added with anticoagulants, the infusion of autologous blood during the positioning process should be conducted promptly to prevent blocking the positioning needle tip and increasing the number of positioning punctures. All patients experienced good localization under local anesthesia, no hemoptysis, and no evident pain after localization.

Logistics analysis suggested that the puncture depth into the lung and the nodule size are independent risk factors for pneumothorax. A deep puncture depth into the lung and a small nodule size increased the possibility of the occurrence of pneumothorax. The reasons may be as follows: ① When the distance between the lesion and the pleura is long, the puncture needle passes through more tissues, increasing the severity of the induced injury. Ineffective control of the injection depth causes the injection direction to be adjusted repeatedly, increasing the possibility of pneumothorax. ② Small lesions are greatly affected by the patient’s respiratory movement during puncture and are easy to deviate from the target. The area under the ROC curve was 0.713, and after further internal validation with bootstrap 1000, the area under the ROC curve was 0.713 (0.6026–0.7196), indicating that the ability of this model to distinguish between patients with pneumothorax after positioning with this technology was acceptable.

The risk factors for the diameter of pulmonary nodules could not be changed in this study. Compared with it, shortening the distance of the puncture needle into lung tissue is a feasible and effective strategy. The judgment of the location of pulmonary nodules mainly depends on the blood tattoos formed on the pleural surface of the visceral layer of the lung tissue after localization by autologous blood, thus reducing the circumference of target nodules palpated by fingers in VATS. After the initial confirmation of the location of the pulmonary nodule, the marked point was sutured with a thread crochet. After taking out the resected lung tissue specimen, carefully palpating the fingers according to the marked point to find the lesion, using the hook needle again to suture the target nodule, sending the rapid frozen pathological section during the operation to the pathologist for pathological diagnosis, but injecting about 5–10 mm of autologous blood around the target nodule has little effect on this process.

CT-guided injection of autologous blood as a novel localization technology for PNs was underreported both locally and internationally. It is convenient, simple and effective, economical and practical, can effectively reduce costs, has fewer side effects and complications, and can improve the success rate of thoracoscopic pulmonary wedges. However, reports on risk factors related to pneumothorax after localization using this technique were not investigated. In this study, smaller nodules and deeper contact between the needle and lung tissue were related to pneumothorax after CT-guided localization using autologous blood. The predictive model provides a useful tool for assessing the risk of PNs with pneumothorax owing to autologous blood localization and may help patients and clinicians select the best risk reduction strategy based on individual risks, which has certain significance for clinical guidance and diagnosis.

Our study had limitations because it lacked randomness and was a retrospective analysis conducted at a single site, making it susceptible to potential bias. In addition, the sample size of this study is relatively small and might have influenced the conclusion of the safety and effectiveness of the autologous blood localization method; therefore, it may need to be studied in a larger population size.

## Conclusions

In conclusion, The factors associated with pneumothorax after localization are smaller nodules and deeper contact between the needle and lung tissue.

## Data Availability

No datasets were generated or analysed during the current study.

## Abbreviations

CT: Computed tomography
PN: Pulmonary nodule
VATS: Video-assisted thoracoscopic surgery
FVC: Forced vital capacity
FEV1/FVC: Forced expiratory volume in the first second/forced vital capacity
MVV: Maximal voluntary ventilation
WBC: White blood cell
GGN: Ground-glass nodule
